# New Meroterpenoids from the Endophytic Fungus *Aspergillus flavipes* AIL8 Derived from the Mangrove Plant *Acanthus ilicifolius*

**DOI:** 10.3390/md13010237

**Published:** 2015-01-07

**Authors:** Zhi-Qiang Bai, Xiuping Lin, Junfeng Wang, Xuefeng Zhou, Juan Liu, Bin Yang, Xianwen Yang, Shengrong Liao, Lishu Wang, Yonghong Liu

**Affiliations:** 1 Key Laboratory of Tropical Marine Bio-resources and Ecology, Guangdong Key Laboratory of Marine Materia Medica, RNAM Center for Marine Microbiology, South China Sea Institute of Oceanology, Chinese Academy of Sciences, Guangzhou 510301, China; E-Mails: baizhiqiang10@mails.ucas.ac.cn (Z.-Q.B); xiupinglin@hotmail.com (X.L.); Junfeng1982a@163.com (J.W.); xfzhou@scsio.ac.cn (X.Z.); ljuan2010@qq.com (J.L.); bingo525@163.com (B.Y.); xwyang@scsio.ac.cn (X.Y.); ljrss@126.com (S.L.); 2 State Key Laboratory of Phytochemistry and Plant Resources in West China, Kunming Institute of Botany, Chinese Academy of Sciences, Kunming 650204, China; 3 Jilin Provincial Academy of Chinese Medicine Sciences, Changchun 130021, China; E-Mail: wls6856@163.com

**Keywords:** meroterpenoid, *Aspergillus flavipes*, endophytic fungus

## Abstract

Four new meroterpenoids (**2**–**5**), along with three known analogues (**1**, **6**, and **7**) were isolated from mangrove plant *Acanthus ilicifolius* derived endophytic fungus *Aspergillus flavipes*. The structures of these compounds were elucidated by NMR and MS analysis, the configurations were assigned by CD data, and the stereochemistry of **1** was confirmed by X-ray crystallography analysis. A possible biogenetic pathway of compounds **1**–**7** was also proposed. All compounds were evaluated for antibacterial and cytotoxic activities.

## 1. Introduction

Marine-derived fungi have recently become a hotspot of new and bioactive metabolites in the marine environment [1]. The mangrove plant *Acanthus ilicifolius* growing in tropical and subtropical intertidal habitats is a rich source of new natural products. The genus *Aspergillus* is one of the major contributors to the secondary metabolites of fungal origin. Cytotoxic cytochalasins have been isolated from endophytic fungus *Aspergillus flavipes* associated with *A. ilicifolius*. Cytochalasins have the ability to bind to actin filaments and block polymerization and the elongation of actin [2]. Spiroquinazolines [3], cerebrosides [4], isobenzofurans [5,6], cytochalasins [2,7,8,9], and butyrolactones [10] have been isolated from the culture of *A. flavipes*. They showed antibiotic [9,11,12], cytotoxic [7], substance-p inhibitory [3], peptide deformylase inhibitory [5,6], and HIV-1 integrase inhibitory activities [8].

**Figure 1:**
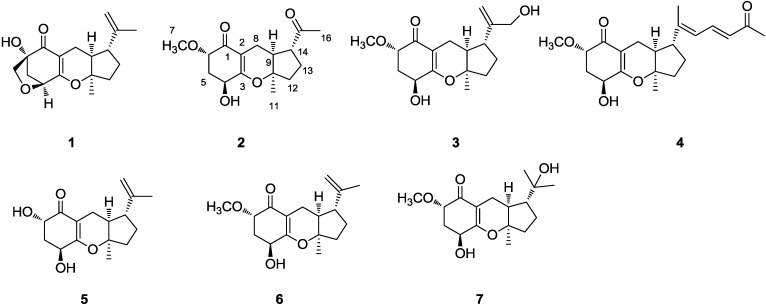
The chemical structures of compounds **1**–**7**.

In the course of our study on marine derived microbes, several active metabolites were isolated [13,14,15,16]. In our previous study, two aromatic butyrolactones were reported from the same fungus, *A. flavipes* [17]. In a continuing study, four new and three known meroterpenoids were isolated from the same fungus. Herein, the isolation, structural elucidation, biological activities, and plausible biogenetic pathway of compounds **1**–**7** are reported (Figure 1).

## 2. Results and Discussion

### 2.1. Structure Elucidation

**Figure 2:**
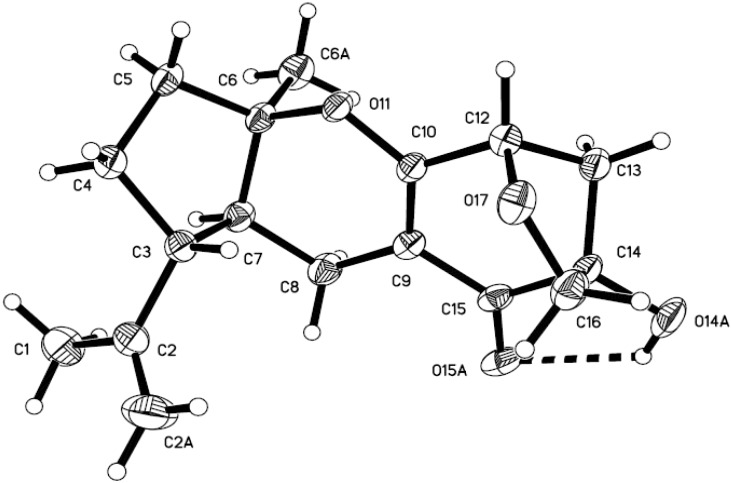
The X-ray structure of compound **1**.

Compound **1** was isolated as colorless crystals. The molecular formula of **1** was established as C_17_H_22_O_4_ according to MS and NMR data. The NMR and optical rotation data were in agreement with those previously published, guignardone A [18]; the absolute configuration was confirmed by X-ray crystallography (Figure 2).

Compound **2** was obtained as yellow oil. Its molecular formula was determined to be C_16_H_22_O_5_ by HRESIMS [M + Na]^+^ 317.1363 (calcd for 317.1359) and [M + H]^+^ 295.1544 (calcd for 295.1540). The ^1^H and ^13^C NMR spectra of **2** were similar to guignardone H (**6**) [19], with the only obvious difference being the presence of a ketone group (IR ν_max_ 1608, δ_C_ 212.0) instead of a terminal double bond and the presence of a methyl ketone singlet downfield replacing the original vinyl methyl singlet observed in guignardone H. A small coupling constant of 3.0 Hz between H-6 and H-5 placed H-6 in a pseudoequatorial orientation of the cyclohexanone ring. Hence, H-6 was deduced to be β-oriented. Since the specific rotation and CD spectrum of compound **2** has the same sign as guignardone A (**1**) [18], and on biogenetic grounds, compound **2** was tentatively assigned as having the same absolute configuration as guignardone A.

**Figure 3:**
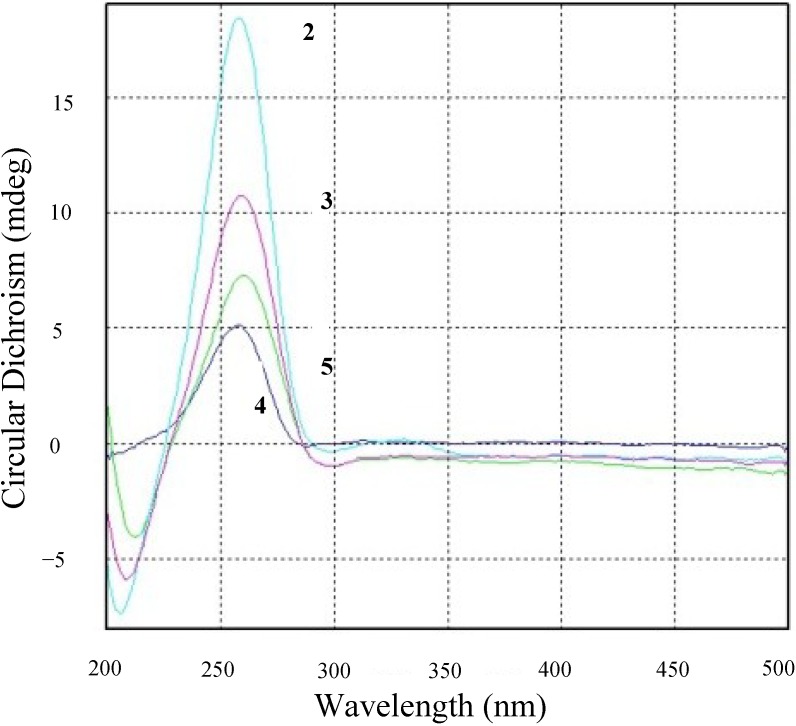
Circular Dichroism profiles of compounds **2**–**5**.

Compound **3** was obtained as a colorless oil. Its molecular formula was determined as C_17_H_24_O_5_ by HRESIMS [M + Na]^+^ 331.1516 (calcd for 331.1521) and [M + H]^+^ 309.1697 (calcd for 309.1702). IR absorptions implied the presence of hydroxyl group. The ^1^H and ^13^C NMR spectra of **3** were similar to guignardone H (**6**) [19], with the only obvious difference being the presence of a methyl group replacing a hydroxymethylene group (δ_C_ 65.3). A small coupling constants of 3.0 Hz between H-6 and H-5 indicated β-oriented of H-6. The configuration of **3** was tentatively established as same as that of **2** for the biogenetic pathway consideration. The optical rotation exhibited the same sign with **2**, and the CD spectrum of **3** showed the same profile with **2** (Figure 3), and thus absolute stereochemistry of **3** was the same as **2**, and named guignardone K (**3**).

Compound **4** was isolated as a yellow oil. Its molecular formula was determined as C_21_H_28_O_5_ by HRESIMS [M + Na]^+^ 383.1835 (calcd for 383.1834) and [M + H]^+^ 361.2012 (calcd for 361.2015). IR absorptions implied the presence of a hydroxyl, a ketone, and a conjugated double bond groups. The ^13^C NMR and DEPT spectra (Table 1) revealed 21 carbon signals, including four methyl (δ_C_ 14.4, 22.2, 27.7, and 58.5), four methylene (δ_C_ 16.2, 26.9, 34.4, and 37.7), seven methine (one oxygenated), and six quaternary carbon (including two α, β-unsaturated carbonyl and two oxygenated). Comparison of the ^1^H NMR and ^13^C NMR spectroscopic data with guignardone H (**6**) [19] revealed that **4** had the same tricyclic moiety as **6**, but different side chain. The geometry of the disubstituted double bond (C-18) was determined to be *E* on the basis of the large coupling constants of the respective protons (*J* = 15.0 Hz). The presence of a methyl ketone group (δ_H_ 2.27, s; δ_C_ 198.7 and 27.7) at the end of the side chain of **4**, which was confirmed by the HMBC correlations, the side chain was connected to the tricyclic moiety at C-5 in the ring. The absolute configuration of **4** was assumed to be the same as that of **2**, and named guignardone L (**4**).

Compound **5** was isolated as a yellow oil. Its molecular formula was determined as C_16_H_23_O_4_ by HRESIMS [M + Na]^+^ 301.1407 and [M + H]^+^ 279.1590. Comparison of the ^1^H NMR and ^13^C NMR spectroscopic data with guignardone G [19], revealed that **5** had the same structure as guignardone G, but different stereochemistry at C-6. H-6 was deduced to be β-oriented by a small coupling constant of 3.0 Hz between H- 6 and H-5. The configuration of **5** was established as same as that of **2**, and named guignardone M (**5**).

Compounds **6** and **7** were identified as guignardones H [19] and I [19,20], respectively, by comparison of the ^1^H and ^13^C NMR with those reported data.

### 2.2. Plausible Biosynthetic Pathway

**Figure 4:**
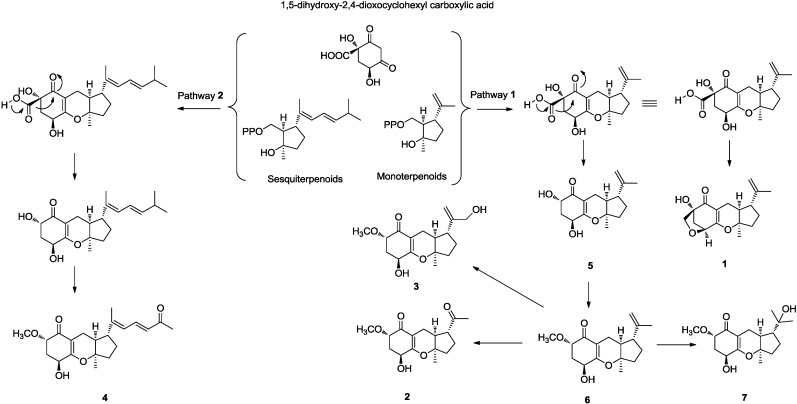
Plausible biosynthetic pathway of compounds **1**–**7**.

As compounds **1**–**7** were co-metabolites, we propose that they are on the same biosynthetic pathway. A possible biogenetic pathway of compounds **1**–**7** was proposed as shown in Figure 4. As suggested in pathways 1 and 2, we hypothesized the alkylation of precursor 1,5-dihydroxy-2,4-dioxocyclohexyl carboxylic acid by monoterpenoids or sesquiterpenoids to give the intermediates. Further modification leads to the generation of compounds **1**–**7**.

**Table 1:** ^1^H and ^13^C NMR data of compounds **2**–**5** (500/125 MHz).

	2		3		4		5	
	^1^H	^13^C	^1^H	^13^C	^1^H	^13^C	^1^H	^13^C
1		198.6 s		194.9 s		194.9 s		198.0 s
2		106.2 s		105.7 s		105.7 s		104.2 s
3		171.9 s		166.7 s		167.8 s		169.0 s
4	4.54 brt 5.0	66.4 d	4.28 brt 5.0	65.8 d	4.26 t 5.0	65.7 d	4.55 t 5.5	65.4 d
5	1.90 m	37.4 t	2.30 m	34.5 t	1.96 m	34.4 t	2.12 m	38.2 t
	2.64 m		2.41 m		2.40 m		2.28 m	
6	3.88 dd 5.0, 3.0	79.5 d	3.75 dd, 5.0, 3.0	79.2 d	3.73 dd 5.0, 3.0	79.2 d	4.09 dd 5.5, 3.0	69.4 d
7	3.53 s	58.5 q	3.47 s	58.4 q	3.50 s	58.5 q		
8	2.28 m	18.6 t	2.35 m	16.3 t	2.31 m	16.2 t	2.26 m	16.4 t
			2.19 m					
9	2.46 m	42.8 d	2.12 m	43.8 d	2.06 m	43.5 d	1.96 m	43.3 d
10		89.6 s		87.6 s		87.5 s		89.0 s
11	1.36 s	22.4 q	1.35 s	22.4 q	1.36 s	22.2 q	1.35 s	23.1 q
12	1.90 m	38.7 t	1.88 m	37.3 t	2.00 m	37.7 t	1.89 m	37.6 t
	2.22 m		2.15 m		2.25 m		2.15 m	
13	1.82 m	26.1 t	2.06 m	28.4 t	1.84 m	26.9 t	1.59 m	26.9 t
	2.20 m		1.57 m		2.30 m		1.95 m	
14	2.68 m	55.4 d	2.27 m	44.8 d	2.37 m	51.4 d	2.16 m	49.1 d
15		212.0 s		149.7 s		150.2 s		145.3 s
16			4.88 s	109.9 t	1.85 s	14.4 q	4.74 s	111.5 t
			5.10 s				4.63 s	
17	2.16, s	29.2 q	4.11 d 1.5	65.3 d	5.94 d 10.0	124.7 d	1.67 s	19.2 q
18					7.41 dd 15.0, 10.0	138.8 d		
19					6.09 d 15.0	129.3 d		
20						198.7 s		
21					2.27 s	27.7 q		

### 2.3. Discussion

All isolated compounds were evaluated for antibacterial and cytotoxic activities; none of them are active. The previously reported compounds that have similar molecular architectures with meroterpenoids **1**–**7**, showed antibacterial [19], cytotoxic [2,21,22], antimicrobial [22], and phytotoxic [23,24,25] activities. The structural differences of compounds **1**–**7** mainly occur in the isoprenoid chain, this may be the reason for their lack of activities. Tricycloalternarene-type meroterpenoids have been isolated from fungi *Alternaria citri* [26], *A. alternata* Ly83 [27,28], *A. alternate* [23,24,29,30], *A. tenuissima* [31], *A. tenuissima* SY-P-07 [32], *Guignardia bidwellii* PSU-G11 [21], *G. Mangiferae* [18,33,34], *Septoria* spp. [25], *Ulocladium* sp. [22], and *Pycnoporus sanguineus* [20]. Tricycloalternarene-type metabolites appear to be characteristic of the genera *Alternaria* and *Guignardia*, and thus may be good chemotaxonomic markers for these two genera. This is first time to isolate this class of compounds from fungus *A. flavipes*.

## 3. Experimental Section

### 3.1. General Experimental Procedures

Optical rotations were measured using a Anton Paar MCP-500 Polarimeter. Ultraviolet (UV) spectra were measured in acetone on a Shimadzu UV-2600 Spectrophotometer. Infrared spectra (IR) were recorded on a Shimadzu IR Affinity-1FT-IR Spectrometer. One-dimensional and two-dimensional NMR spectra were recorded on a 500 MHz Bruker FTNMR Spectrometer using TMS as an internal standard. ESIMS and HRESIMS were measured with Bruker maXis impact and amaZon speed Mass Spectrometers. Column chromatography was performed on silica gel (90–150 μm, Qingdao Marine Chemical Company, Qingdao, China), Sephadex LH-20 (40–70 μm, Amersham Pharmacia Biotech AB, Uppsala, Sweden). Column chromatography was performed with silica gel (100–200 mesh; 300–400 mesh; Jiangyou Silica Gel Development, Inc., Yantai, China) and Sephadex LH-20. Thin layer chromatography (TLC, 0.1–0.2 mm or 0.3–0.4 mm) was carried out with precoated silica gel plates (GF-254, Jiangyou Silica Gel Development, Inc., Yantai, China). TLC spots were visualized under UV light and heating after spray by 5% H_2_SO_4_ in EtOH.

### 3.2. Fungal Material

The endophytic fungus *A. flavipes* AIL8 was isolated from the inner leaves of mangrove plant *A. ilicifolius* collected at Daya Bay, Shenzhen, China, in 2011. This fungus was deposited at 4 °C on potato dextrose agar (PDA) slants in CAS Key Laboratory of Tropical Marine Bio-resources and Ecology, South China Sea Institute of Oceanology, Chinese Academy of Sciences, Guangzhou, China.

The strain stored on PDA slants was inoculated and cultured on PDA agar plates for 7 days. Seed medium (potato 200 g, dextrose 20 g, distilled water 1000 mL) in 100 mL × 10 Erlenmeyer flasks was inoculated with fungus and incubated at 25 °C for 48 h on a rotating shaker (180 rpm). Production medium of solid rice in 1000 mL flasks (rice 200 g, distilled water 210 mL) was inoculated with seed solution (10 mL) one for one. Flasks were incubated at 25 °C under still culture and fermented for 40 days, cultures from 30 flasks were harvested for the isolation of substances. Fungal identification was carried out by using the method previously reported [13].

### 3.3. Extraction and Isolation

After fermentation, culture medium containing the mycelium was inactivated by acetone and extracted with ethyl acetate, and evaporated (40 °C) to remove EtOAc. About 180 g residue was obtained, the residue was dissolved and using the similar procedure as previously described [35], and got 99.0 g residue to chromatograph on silica gel and eluted with solutions gradients, petroleum ether (60–90 °C)/acetone (10:0, 9:1, 8:2, 7:3, 6:4, 5:5), chloroform/methanol (10:0, 9:1, 8:2, 7:3, 6:4, 5:5, 0:1), to yield 9 fractions (Frs. 1–9). Frs. 2 chromatographed on a Sephadex LH-20 column using CHCl_3_/MeOH (1:1), to produce 4 fractions (F_2-1_–F_2-4_), was further separated by silica gel column chromatography and eluted with (petroleum ether/acetone, 15:1), to get 4 fractions (F_2-1_–F_2-4_). F_2-2_ was applied to silica gel eluted with a gradient of (petroleum ether/acetone, 18:1) to give **1** (50 mg). Frs. 3 chromatographed on a Sephadex LH-20 column using CHCl_3_/MeOH (1:1), to produce 7 fractions F_3-1_–F_3-7_. F_3-2_ on a silica gel column (petroleum ether/acetone, 10:1) to produce 6 fractions, the second fraction was chromatographed on silica gel (petroleum ether/acetone, 15:1) to give **6** (11.3 mg). Frs. 4 chromatographed on a Sephadex LH-20 column using CHCl_3_/MeOH (1:1), to produce 5 fractions (F_4-1_–F_4-5_), F_4-1_- F_4-3_ was chromatographed on a silica gel column (petroleum ether/acetone, 10:1) to produce compounds **2** (6.6 mg), **3** (7.1 mg), and **7** (5.6 mg). Frs. 5 chromatographed on a Sephadex LH-20 column using CHCl_3_/MeOH (1:1), to produce 3 fractions (F_5-1_–F_5-3_), F_5-2_ was further separation on a silica gel column (petroleum ether/acetone, 8:1) to give compounds **4** (7.7 mg) and **5** (9.4 mg).

Guignardone J (**2**). Yellow oil; ${\left[\alpha \right]}_{D}^{25}$ +35 (*c* 0.66, acetone); UV (MeOH) λ_max_: 325 nm (3.25); IR (KBr) ν_max_ 2924, 1608, 1165, 1076 cm^−1^; ^1^H and ^13^C NMR data, see Table 1; HRESIMS *m/z* 317.1363 [M + Na]^+^ (calcd for C_16_H_22_O_5_Na, 317.1365); HRESIMS [M + H]^+^ 295.1544 (calcd for C_16_H_23_O_5_, 295.1545).

Guignardone K (**3**). Colorless oil; ${\left[\alpha \right]}_{D}^{25}$ +100 (*c* 0.7, acetone); UV (MeOH) λ_max_: 327 (2.89), 306 nm (1.43); IR (KBr) ν_max_ 3390, 2927, 1284, 1076 cm^−1^; ^1^H and ^13^C NMR data, see Table 1; HRESIMS *m/z* 331.1516 [M + Na]^+^ (calcd for C_17_H_24_O_5_Na, 331.1521), *m/z* 309.1697 [M + H]^+^ (calcd for C_17_H_25_O_5_, 309.1702).

Guignardone L (**4**). Yellow oil; ${\left[\alpha \right]}_{D}^{25}$ +50 (*c* 0.5, acetone); UV (MeOH) λ_max_: 327 (3.05), 306 nm (1.56); IR (KBr) ν_max_ 3417, 1612 cm^−1^; ^1^H and ^13^C NMR data, see Table 1; HRESIMS *m/z* 383.1835 [M + Na]^+^ (calcd for C_21_H_28_O_5_ , 383.1834), [M + H]^+^ 361.2012 (calcd for C_21_H_29_O_5_, 361.2015).

Guignardone M (**5**). Yellow oil; ${\left[\alpha \right]}_{D}^{25}$ +60 (*c* 0.5, acetone); UV (MeOH) λ_max_: 360 (3.18), 324 nm (2.04); IR (KBr) ν_max_ 3352, 1608, 1141, 1060 cm^−1^; ^1^H and ^13^C NMR data, see Table 1; HRESIMS [M + Na]^+^ 301.1407 (calcd. for C_16_H_22_O_4_Na, 301.1416), [M + H]^+^ 279.1590 (calcd for C_16_H_23_O_4_, 279.1596).

### 3.4. X-ray Crystallographic Analysis of Guignarenone A (**1**)

Colorless crystal of C_17_H_22_O_4_, M = 290.35, monoclinic, C2, a = 19.5512(2) Å, b = 5.60488(7) Å, c = 14.50891(19) Å, V = 1512.61(3) Å^3^, Z = 4, *D*_calc_ = 1.275 g/cm^3^, F(000) = 624, crystal size: 0.43 × 0.38 × 0.29 mm^3^, independent reflections: 2659 [R(int) = 0.0504], final R indicates [I > 2*σ*(I)]: R1 = 0.0402, wR2 = 0.1018. A total of 16,279 unique reflections were collected using a CrysAlis PRO CCD area detector diffractometer with graphite monochromated Cu Kα radiation (λ = 1.54178Å) at 150 K, Absorption corrections were done by semiempirical from equivalents. The structure was solved using direct methods (SHELXL-97) and refined with Full-matrix least-squares on F^2^, 1 restraint and 195 variable parameters.

### 3.5. Antimicrobial Activity

Antibacterial activity was tested on strains of *Staphylococcus aureus*, *Escherichia coli*, *Bacillus subtilis*, *Bacillus thuringiensis*, *Candida albicans*, and *Micrococcus luteus*. MIC values of the compounds were determined by the modified 0.5 Mcfarland standard method. Two-fold dilutions of the compounds in the range of (40–0.31 mg/mL) were prepared in 0.5% MeOH. Compounds and positive control (Penicillin) were similarly diluted in 0.5% MeOH to generate a series of concentration, ranging from 40 to 0.31 mg/mL per well. The turbidity of the bacterial suspensions was measured at 600 nm, and adjusted with a medium to match the 0.5 McFarland standard (10^5^–10^6^ colony forming units/mL). Subsequently, 180 μL of bacterial culture was inoculated into each well, and the test solutions (20 μL) were added to 96-well plates. Finally, the plates were incubated at 36 °C for 24 h, and the MIC values were determined in triplicates and re-examined at appropriate times. To ensure that these vehicles had significant effect on the bacterial growth, each of the bacterial species was additionally cultured in a blank solution containing LB broth media at concentrations equivalent to those of test solutions [36].

### 3.6. Cytotoxicity

Proliferation of PC-3 cells was evaluated by using a cell counting kit (CCK-8, Ddojindo, Japan) following the manufacturer’s protocol. Cells were routinely grown and maintained in mediums RPMI or DMEM with 10% FBS and with 1% penicillin/streptomycin. All cell lines were incubated in a Thermo/Forma Scientific CO_2_ Water Jacketed Incubator with 5% CO_2_ in air at 37 °C. Cell viability assay was determined by the CCK8 assay. Three-fold dilutions of the compounds in the range of (10–5.08 × 10^5^ μM) were prepared in DMSO. Cells were seeded at a density of 400–800 cells/ well in 96 well plates and treated with various concentration of compounds, positive control (Trichostatin A and Taxol) or solvent control. After 72 h incubation, CCk8 reagent was added, and absorbance was measured at 450 nm using Envision 2104 multi-label Reader (Perkin Elmer, USA). Dose response curves were plotted to determine the IC_50_ values using Prism 5.0 (GraphPad Software Inc., San Diego CA, USA). The inhibitory rate was calculated using the formula: (*OD*_control cells_ − *OD*_treated cells_)/*OD*_control cells_ × 100% [37,38].

## 4. Conclusions

Four new meroterpenoids, along with three known analogues, were isolated from marine-derived endophytic fungus *A. flavipes* AIL8 derived from the mangrove plant *Acanthus ilicifolius*. A possible biogenetic pathway of compounds **1**–**7** was proposed. All compounds were evaluated for antibacterial activity and cytotoxicity; none of them are active. Comparison with those reported tricycloalternarenes, the structural differences mainly occur in the isoprenoid side chains, this maybe the reason for their lack of antibacterial and cytotoxic activities.

## Supplementary Information

Electronic supplementary information (ESI) available: 1D and 2D NMR, and HRESIMS spectral data of compounds **1**–**7**.
